# Similar short-term clinical response to high-dose versus low-dose methotrexate in monotherapy and combination therapy in patients with rheumatoid arthritis

**DOI:** 10.1186/s13075-017-1468-9

**Published:** 2017-11-22

**Authors:** Sytske Anne Bergstra, Cornelia F. Allaart, Rosaline van den Berg, Arvind Chopra, Nimmisha Govind, Tom W. J. Huizinga, Robert B. M. Landewe

**Affiliations:** 1 Department of Rheumatology, Leiden University Medical Centre, Leiden, The Netherlands; 2 Department of Rheumatology, Erasmus University Medical Center, Rotterdam, The Netherlands; 3 Center for Rheumatic Diseases, Pune, India; 4 University of the Witwatersrand, Johannesburg, South Africa; 5 Amsterdam Rheumatology & Immunology Center, Amsterdam, The Netherlands; 6 Zuyderland Medical Center, Heerlen, The Netherlands

**Keywords:** Rheumatoid arthritis, Methotrexate, Dose, Outcome measures, Combination therapy

## Abstract

**Background:**

Aiming at rapid decrease of disease activity, there has been a trend to start with higher doses of methotrexate (MTX) in patients newly diagnosed with rheumatoid arthritis (RA), both as monotherapy and in combination with other antirheumatic drugs. We aimed to study the relationship between clinical response and MTX dose as monotherapy or combination therapy in patients with early RA.

**Methods:**

Disease-modifying anti-rheumatic drug (DMARD)-naive patients with early RA, from a large international observational database, the METEOR database, were selected if MTX was part of their initial treatment. Patients were divided into four groups: MTX monotherapy, MTX + convention synthetic (cs)DMARDs, MTX + glucocorticoids or MTX + biologic (b)DMARDs. MTX dose was dichotomized: low dose ≤10 mg/week; high dose ≥15 mg/week. Linear mixed model analyses for the Disease Activity Score (DAS), DAS in 28 joints (DAS28) and Health Assessment Questionnaire (HAQ) were performed in each medication group, with MTX dose and time as covariates. Outcomes were assessed from baseline until 3–6 months follow up. Associations were adjusted for potential confounding by indication using propensity score (PS) modelling.

**Results:**

For patients starting MTX monotherapy (*n* = 523), MTX + csDMARDs (*n* = 266) or MTX + glucocorticoids (*n* = 615), the PS-adjusted effects of MTX dose (high versus low) on the DAS, DAS28 and HAQ were small and not clinically meaningful. Patients starting MTX + bDMARDs were disregarded due to low numbers (*n* =11).

**Conclusions:**

In patients newly diagnosed with RA, no clinical benefit of high compared to low initial MTX doses was found for MTX monotherapy or for MTX combination therapy with csDMARDs or glucocorticoids.

**Electronic supplementary material:**

The online version of this article (doi:10.1186/s13075-017-1468-9) contains supplementary material, which is available to authorized users.

## Background

Methotrexate (MTX) is the anchor drug in the treatment of rheumatoid arthritis (RA). Current recommendations for MTX monotherapy suggest initiation of 15 mg/week orally, and escalation with 5 mg/month to 25–30 mg/week or the highest tolerable dose [1, 2]. There are no specific recommendations for use of MTX in combination with other antirheumatic drugs (glucocorticoids, conventional synthetic disease-modifying antirheumatic drugs (csDMARDs) and/or biologic DMARDs (bDMARDs)). Many studies have shown faster reduction of disease activity, quicker improvement in physical functioning and less radiographic evidence of damage progression on MTX combination therapy than on MTX monotherapy [3–6]. It is questionable whether a higher initial MTX dose in combination with other effective medication is more effective in the short term than a lower initial MTX dose. The CONCERTO study compared four treatment arms with different MTX doses (2.5, 5, 10 or 20 mg/week) in combination with adalimumab 40 mg/2 weeks in patients with early RA [7]. More patients achieved the Disease Activity Score in 28 joints (DAS28) low disease activity or remission status with increasing MTX doses over 26 weeks. However, radiographic progression and the Health Assessment Questionnaire (HAQ) scores were similar in the various arms. The proportions of patients achieving low disease activity or remission were similar in the MTX 10 mg/week and MTX 20 mg/week arms.

Recently, a meta-regression analysis of trials in patients with recent onset RA showed that higher initial MTX doses were not associated with better short-term clinical outcomes, neither with MTX monotherapy, nor with MTX in combination with bDMARDs or glucocorticoids [8].

In the current study we aimed to assess the influence of MTX dose on disease outcomes and physical functioning in an international cohort with real-life data. We hypothesized that in patients newly diagnosed with RA the initial MTX dose as monotherapy or in combination with other csDMARDs, bDMARDs or glucocorticoids will not determine short-term outcomes.

## Methods

### Data selection

Data from the international, observational, Measurement of Efficacy of Treatment in the Era of Outcome in Rheumatology (METEOR) database were used, which has been described previously [9]. For the current study, we selected all DMARD-naive patients with early RA and symptom duration <5 years, with ≥ 1 follow-up visits after 3–6 months. At both baseline and follow-up visits, patients had to have at least one of the following outcome measures: DAS, DAS28, erythrocyte sedimentation rate (ESR), C-reactive protein (CRP) or HAQ. MTX had to be part of the initial treatment as monotherapy or in combination with other csDMARDs/bDMARDs/glucocorticoids. Variation in dose was allowed (e.g. step-up MTX dose or step-down prednisone dose) but no change in medication type was allowed between initial treatment and the follow-up visit after 3–6 months. Since the METEOR database consists of observational data gathered in clinical practice, there are irregular time intervals between follow-up visits and the number of follow-up visits differs per patient. Therefore, the last visit within 3–6 months after treatment initiation, meeting all inclusion and exclusion criteria was defined for each patient, and all follow-up visits between baseline and this last follow-up visit were selected. In order to take into account step-up dosing schedules, the MTX dose prescribed at the final visit before 3 months follow up was used.

### Statistical analysis

Patients were analysed in four groups, based on initial MTX strategy: (1) MTX monotherapy, (2) MTX + other csDMARDs, (3) MTX + glucocorticoid (+/− additional csDMARDs) or (4) MTX + bDMARD (+/− additional csDMARDs). Missing data were imputed using multivariate normal multiple imputation (30 cycles). Linear mixed model (LMM) analyses were performed to assess the effectiveness of MTX dose on the outcome measures DAS, DAS28 and HAQ, within the four groups. To account for irregular time intervals, random intercept and slope were added to each model, with “independence” covariance matrix. MTX dose was dichotomized (low dose ≤10 mg/week; high dose ≥15 mg/week). Time in days between baseline and each follow-up visit was added as continuous variable.

Differences in environmental and patient characteristics may affect the initial MTX dose, and therefore may have caused confounding by indication. To adjust for potential confounding, the propensity score (PS) was calculated in the imputed dataset, using multiple probit regression analysis based on observed baseline patient and environmental characteristics [10]. Several PS models were tested and compared for the best data fit in all 30 imputations. Representing the probability of receiving an intervention given the observed baseline variables, the PS was then added as covariate adjustment to the LMM analyses. Details on the PS are given in Additional file 1. All LMM analyses were performed with and without PS, to see whether confounding by indication was present. All analyses were performed using STATA SE 14 (StataCorp LP).

## Results

**Fig. 1:**
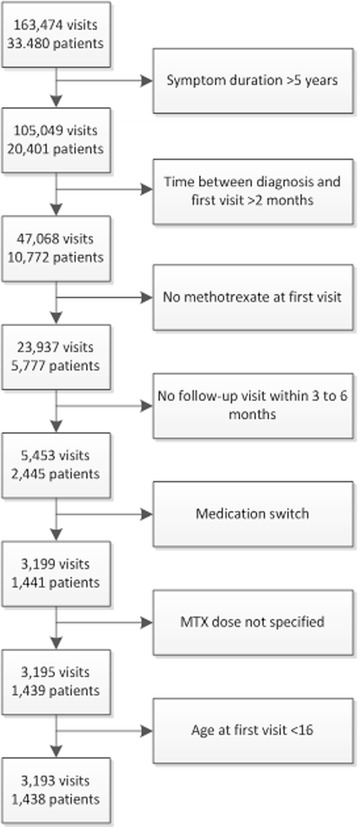
Flow chart of the patient selection

From the METEOR database, 1438 patients (3193 visits) were selected: 523 patients (1120 visits) started MTX monotherapy, 266 patients (581 visits) started MTX + csDMARDs, 615 patients (1416 visits) started MTX + glucocorticoids and 11 patients (26 visits) started MTX + bDMARDs (Fig. 1). Detailed information on concomitant treatment is presented in Additional file 2. Patients originated from 20 different countries, with 94% of data originating from India, South-Africa, Portugal, the Netherlands, the USA, Ireland and Mexico. Too few patients started MTX + bDMARDs to perform meaningful analyses. In addition, 23 patients (50 visits) who started MTX 12.5 mg/week (the intermediate dose) were disregarded. Baseline characteristics of the other patients are shown in Table 1. There was a trend over time to start higher MTX doses (Additional file 3).

**Table 1:** Baseline characteristics per treatment group, non-imputed data

	MTX monotherapy (*n* = 523)		MTX + csDMARDs (*n* = 266)		MTX + glucocorticoids (*n* = 615)	
	Number		Number		Number	
Age at first visit (years)	522	47.9 (13.1)	264	44.6 (10.9)	479	48.3 (14.8)
Gender (% female)	520	78	266	83	609	81
Body mass index	281	26.6 (6.7)	184	27.6 (6.3)	272	27.2 (6.0)
Symptom duration at diagnosis median (IQR)	451	365 (169–731)	266	730 (365–1095)	482	458 (181–1095)
Rheumatoid factor (% positive)	511	77	263	84	585	81
ACPA (% positive)	300	72	98	85	342	76
Erosions present (% positive)	305	40	62	55	293	52
ESR	462	56.5 (33.0)	241	69.3 (31.7)	543	59.5 (35.5)
CRP	415	33.1 (33.9)	219	40.3 (35.5)	515	37.7 (37.1)
HAQ	439	1.0 (0.6)	249	1.1 (0.6)	506	1.3 (0.7)
DAS	314	3.7 (1.2)	189	4.0 (0.96)	347	3.9 (1.2)
DAS 28	340	5.7 (1.5)	192	6.2 (1.2)	415	6.0 (1.5)
MTX dose (% high dose)	523	28	266	14	615	46
Follow-up duration (days)	523	134 (28)	266	135 (28)	615	139 (31)

Data per number of patients are means (SD), unless indicated otherwise. *MTX* methotrexate, *csDMARDs* conventional synthetic disease-modifying antirheumatic drugs, *IQR* inter quartile range, *ACPA* anti-citrullinated protein antibodies, *ESR* erythrocyte sedimentation rate, *CRP* C-reactive protein, *HAQ* Health Assessment Questionnaire, *DAS* Disease Activity Score

Because physicians were free to choose their own disease activity measure, the DAS and DAS28 based on ESR were missing in 40% and 35% of all visits, respectively. However, an official disease activity measure was only unavailable in 4% of all visits and a disease activity measure component was only unavailable in 0.3% of all visits.

In Table 2, the PS-adjusted and unadjusted coefficients for the association between initial MTX dose and outcomes within 3–6 months follow-up are presented, stratified by treatment group. For patients starting MTX monotherapy, MTX + csDMARDs or MTX + glucocorticoids, the PS-adjusted effects of MTX dose (high vs low) on DAS, DAS28 and HAQ were small and not clinically meaningful. For example, in the MTX monotherapy group, β (95% CI) for the outcome DAS was 0.070 (−0.15; 0.29), indicating an increase in DAS of 0.070 for a high versus a low MTX dose.

**Table 2:** Unadjusted and propensity score-adjusted results of the linear mixed model analyses to investigate the effectiveness of high versus low methotrexate doses on disease activity (DAS and DAS28) and physical functioning (HAQ), stratified per medication group

	DAS β (95% CI)	DAS28 β (95% CI)	HAQ β (95% CI)
Methotrexate monotherapy (number of patients = 522, number of visits = 1090)			
MTX dose group PS-adjusted	0.070 (−0.15; 0.29)	0.12 (−0.19; 0.43)	0.060 (−0.09; 0.21
MTX dose group unadjusted	−0.63 (−0.79; −0.47)	−0.90 (−0.13; −0.67)	0.16 (0.055; 0.26)
Methotrexate + csDMARDs (number of patients = 262, number of visits = 567)			
MTX dose group PS-adjusted	0.051 (−0.23; 0.33)	0.024 (−0.37; 0.42)	−0.0058 (−0.20; 0.19)
MTX dose group unadjusted	−0.18 (−0.44; 0.072)	−0.28 (−0.63; 0.072)	0.092 (−0.085; 0.27)
Methotrexate + oral glucocorticoid (+/−csDMARDs) (number of patients = 615, number of visits = 1403)			
MTX dose group PS-adjusted	−0.047 (−0.26; 0.16)	−0.16 (−0.44; 0.12)	−0.028 (−0.16; 0.11)
MTX dose group unadjusted	−0.42 (−0.56; 0.28)	−0.74 (−0.93; −0.55)	0.13 (0.045; 0.22)

Time is modelled in days between the baseline visit and each follow-up visit. Low dose is the reference category. MTX dose group is a binary variable with low dose ≤10 mg/week and high dose ≥15 mg/week. *DAS* Disease Activity Score, DAS 28 Disease Activity Score in 28 joints, *HAQ* Health Assessment Questionnaire, *PS* propensity score, *95% CI* 95% confidence interval, *MTX* methotrexate, *csDMARDs* conventional synthetic disease-modifying antirheumatic drug

The unadjusted main associations between MTX dose and outcomes were often in an opposite direction and/or much larger than the PS-adjusted associations, suggesting that confounding by indication indeed plays a role and that it has been (at least partly) corrected for by adjusting for the PS. Two sensitivity analyses were performed: one excluding the country, which added most patients to the analyses (India) and one excluding all patients with a symptom duration >2 years; both resulted in similar outcomes (data not shown).

## Discussion

In this study based on daily practice treatment decisions in patients newly diagnosed with RA, we did not identify clinical benefit of high compared to low MTX starting doses in monotherapy or in combination with csDMARDs or glucocorticoids: high initial MTX doses did not result in greater improvement in the DAS, DAS28 or HAQ compared to low initial MTX doses. Co-medication with csDMARDs or glucocorticoids did not influence this effect. In an earlier meta-regression analysis we showed that also in clinical trials there was no early clinical benefit of a high compared to a low MTX starting dose [8].

We observed a trend over time in daily practice to start higher MTX doses. In particular patients receiving co-medication with glucocorticoids as initial treatment were prescribed higher MTX doses, possibly as the rheumatologist estimated their RA to be more severe. Although we used PS to adjust for baseline differences that may have influenced the treatment decisions of the rheumatologist as well as the outcomes, intangible or unmeasured baseline differences may still affect the results.

We assessed response to treatment within 3–6 months, since current recommendations advise a treat-to-target strategy in which medication is intensified or changed as soon as possible if treatment is not effective. The more rapid onset of action of glucocorticoids as co-treatment may mask any effect of the initial dose of slow-acting MTX [3, 11]. As demonstrated in clinical trials, this appears also to be true for initial treatment with bDMARDs and MTX, but as this is a rare initial treatment in daily practice, we were unable to investigate this further. However, also with MTX monotherapy a higher dose was not more effective than a low dose. The most likely explanation is in the pharmacokinetics of MTX, where stable availability of active MTX polyglutamates seems independent of the weekly MTX dose [12].

This study has potential limitations. The effect of MTX dose was assessed within three subgroups depending on the use and type of co-medication, but within each group, variations in type, number and dose of additional drugs in individual patients could influence efficacy. However, previous clinical trials have shown comparable disease outcomes of various combination therapies and dosing schedules for many drugs are fixed [13, 14]. We dichotomized MTX dosage, and defined MTX >15 mg/week as high dose, which is used in current recommendations, but is still an arbitrary cutoff. The results might have been slightly different with other cutoffs. In addition, MTX was mostly administered orally, and uptake can vary between individuals. We have no further data on the number and timing of patients who might have switched to subcutaneous treatment. The results might have been different with subcutaneous administration of MTX. Moreover, although we are unaware of any evidence that the response to methotrexate could differ between the countries included in the analysis, we took into account the potential influence of country on our outcomes and adjusted for potential country differences by adding country to the propensity score.

Since real-world data were used, no formal procedures were taken to control the quality of clinical assessments, which may have led to more noise compared to clinical trial data. However, our data are in line with previous findings [8].

## Conclusion

In conclusion, these real-world data show that in patients newly diagnosed with RA, a higher MTX dose with or without other csDMARD or glucocorticoids does not result in better clinical efficacy after 3–6 months compared to a low dose. This seems to contradict a general trend over time to start with higher MTX doses. Without apparent early benefit, higher initial MTX dosages may introduce more side effects, which may jeopardise drug retention. However, since side effects were not measured in the METEOR database, we could not assess this. On the other hand, starting a low MTX dose may induce delays in suppression of disease activity and in the introduction of additional therapies, as up to 23% of patients have been found to require higher dosage and up to 56% have not been found to achieve low disease activity with MTX [15]. For the moment, our results suggest that although with MTX monotherapy there may be other considerations, rheumatologists should consider a low instead of a high initial MTX dose, in particular when prescribed in combination with other csDMARDs or glucocorticoids, and further modify treatment according to a treat-to-target protocol.

## Additional files

Additional file 1: Extended description of the propensity score estimation. (DOCX 20 kb)

Additional file 2: Detailed information on concomitant treatment. (DOCX 14 kb)

Additional file 3: Frequency of methotrexate doses over time in newly diagnosed patients in the METEOR database. (DOCX 15 kb)

## Abbreviations

bDMARD: Biologic disease-modifying anti-rheumatic drug
CRP: C-reactive protein
csDMARD: Conventional synthetic disease-modifying anti-rheumatic drug
DAS(28): Disease Activity Score in 28 joints
ESR: Erythrocyte sedimentation rate
HAQ: Health assessment questionnaire
LMM: Linear mixed model
METEOR: Measurement of Efficacy of Treatment in the Era of Outcome in Rheumatology
MTX: Methotrexate
PS: Propensity score
RA: Rheumatoid arthritis
