# Supplementary material for: Similar short-term clinical response to high-dose versus low-dose methotrexate in monotherapy and combination therapy in patients with rheumatoid arthritis
Source: Arthritis Res Ther. 2017 Nov 22;19:258. doi: 10.1186/s13075-017-1468-9 (PMC5700534; doi:10.1186/s13075-017-1468-9)
Supplement: Supplementary file 1 — Extended description of the propensity score estimation. (DOCX 20 kb) [file 13075_2017_1468_MOESM1_ESM.docx]

**Additional file 1: Extended description of the propensity score estimation**

Since the METEOR database is an observational cohort, differences in patients and environmental characteristics may determine differences in the MTX starting dose prescribed to a patient, and thus result in confounding by indication. To adjust for this potential confounding, a propensity score (PS) was calculated in the imputed dataset, using a multiple probit regression analysis based on observed baseline patient and environmental characteristics. These variables could (at least in theory) be either related to both the intervention (in this case, high or low MTX dose) and the outcome (disease activity or HAQ) or to the outcome alone[1]. Several PS models were tested and compared regarding the best fit to the data, in each of the 30 imputations. Models were compared based on the pseudo R^2^, the balance of the model, the region of common support and the final number of blocks. One PS model was calculated for the three medication groups combined. To account for differences in the prescription of a certain MTX dose within these medication groups, medication group was added as a variable to the PS model and the interactions between medication group and each of the other variables in the PS model were tested. Representing the probability of receiving an intervention given observed baseline variables, the PS was then added as a covariate adjustment to the LMM analyses. A region of common support was defined, outside of which patients were disregarded for further analysis. This action ensures that there is no perfect predictability for a patient to belong to the high or the low dose MTX group[2]. Since the region of common support slightly varied for each imputation, patients were disregarded from further analysis if they were considered outside the region of common support in more than half of the imputations (>15).

An example of the final PS model in one of the imputations can be found in supplementary table 1. In total over all medications groups, 36 patients were excluded from the analyses, since they were outside the region of common support. Over all imputations, the average region of common support was 0.017 to 0.98, with a final number of 7 blocks.

Table S1: example of the propensity score model in one of the imputations.

|  | **β** | **P** | **95% CI** |
| --- | --- | --- | --- |
| **Medication group^a^** |  |  |  |
| **MTX + csDMARDs** | 0.077 | 0.579 | -0.20; 0.35 |
| **MTX + glucocorticoids** | 0.087 | 0.435 | -0.13; 0.31 |
| **ESR** | 0.0015 | 0.374 | -0.0018; 0.0048 |
| **HAQ** | -0.017 | 0.846 | -0.19; 0.16 |
| **Ritchie Articular Index** | -0.011 | 0.221 | -0.029; 0.0068 |
| **Swollen joint count** | -0.0039 | 0.636 | -020; 0.012 |
| **Rheumatoid factor** | -0.084 | 0.576 | -0.38; 0.21 |
| **Weight** | 0.010 | 0.006 | 0.0028; 0.017 |
| **Height** | 0.029 | <0.001 | 0.019; 0.039 |
| **Gender** | 0.36 | 0.005 | 0.11; 0.61 |
| **VAS physician global** | 0.0046 | 0.129 | -0.0013; 0.011 |
| **VAS patient pain** | 0.0029 | 0.277 | -0.0023; 0.0081 |
| **ACPA** | 0.054 | 0.704 | -0.23; 0.33 |
| **Presence of erosions** | -0.039 | 0.705 | -0.24; 0.16 |
| **Country of hospital^b^** |  |  |  |
| **Spain** | -1.20 | 0.104 | -2.64; 0.25 |
| **Great Britain** | -2.26 | 0.002 | -3.69; -0.84 |
| **Greece** | -2.05 | 0.026 | -3.86; 2.24 |
| **Ireland** | -0.96 | 0.174 | -2.35; 0.42 |
| **India** | -2.04 | 0.002 | -3.36; -0.73 |
| **Italy** | -2.41 | 0.005 | -4.09; -0.73 |
| **Mexico** | -0.24 | 0.740 | -1.63; 1.16 |
| **Netherlands** | -0.71 | 0.291 | -2.04; 0.61 |
| **Portugal** | -0.33 | 0.620 | -1.65; 0.99 |
| **US** | 0.11 | 0.873 | -1.28; 1.50 |
| **South-Africa** | -0.11 | 0.876 | -1.43; 1.22 |
| **Other^c^** | -1.00 | 0.155 | -2.37; 0.38 |
| **Constant** | -4.93 | <0.001 | -7.07; -2.79 |

^a^Reference category MTX monotherapy ^b^Reference category Cyprus. ^c^Countries with a limited number of patients were added together in the category “other”. Pseudo R^2^ model= 0.47

**References**

1. Brookhart MA, Schneeweiss S, Rothman KJ, Glynn RJ, Avorn J, Sturmer T: Variable selection for propensity score models. Am J Epidemiol 2006; 163(12):1149-56.

2. Heckman J, Ichimura H, Smith J, Todd P: Characterizing Selection Bias Using Experimental Data. Econometrica 1998; 66(5):1017-98.
