# Supplementary material for: Similar short-term clinical response to high-dose versus low-dose methotrexate in monotherapy and combination therapy in patients with rheumatoid arthritis
Source: Arthritis Res Ther. 2017 Nov 22;19:258. doi: 10.1186/s13075-017-1468-9 (PMC5700534; doi:10.1186/s13075-017-1468-9)
Supplement: Supplementary file 2 — Detailed information on concomitant treatment. (DOCX 14 kb) [file 13075_2017_1468_MOESM2_ESM.docx]

**Supplementary file 2: detailed information regarding concomitant treatment**

Table 1. Concomitant treatment in combination therapy with csDMARDs.

| Treatment | Number of patients |
| --- | --- |
| MTX + HCQ | 178 |
| MTX + SSZ | 64 |
| MTX + HCQ + SSZ | 21 |
| other | 3 |

Table 2. Concomitant treatment in combination therapy with glucocorticoids.

| Treatment | Number of patients |
| --- | --- |
| MTX + GC | 394 |
| MTX + HCQ + GC | 112 |
| MTX + SSZ + GC | 75 |
| MTX + HCQ + SSZ + GC | 31 |
| other | 3 |
