# Supplementary material for: Similar short-term clinical response to high-dose versus low-dose methotrexate in monotherapy and combination therapy in patients with rheumatoid arthritis
Source: Arthritis Res Ther. 2017 Nov 22;19:258. doi: 10.1186/s13075-017-1468-9 (PMC5700534; doi:10.1186/s13075-017-1468-9)
Supplement: Supplementary file 3 — Frequency of methotrexate doses over time in newly diagnosed patients in the METEOR database. (DOCX 15 kb) [file 13075_2017_1468_MOESM3_ESM.docx]

**Additional file 3**

Figure 1. Frequency of methotrexate doses over time of newly diagnosed patients in the METEOR database. Low MTX dose ≤10 mg/week, high MTX doses ≥15 mg/week. Only patients and methotrexate doses fulfilling inclusion criteria of this study are presented in this figure.
